# A New and Effective Technique in the Endoscopic Treatment of Obesity and Regulation of Diabetes: The Pyloric Revision

**DOI:** 10.7759/cureus.43357

**Published:** 2023-08-12

**Authors:** Murat Kanlioz, Ugur Ekici

**Affiliations:** 1 General Surgery, Flora Transplantation Institute, Istanbul, TUR; 2 General Surgery, Flora Transplantation Institute, İstanbul, TUR

**Keywords:** feeling of fullness, appetite and sateity, endoscopic obesity treatments, diabetes mellitus, pyloric revision, obesity treatment

## Abstract

Aim: This study aimed to investigate the role of the functional structure of the pylorus in obesity and diabetes and to determine the efficacy of a new method, pyloric revision (PR), in the treatment.

Methods: The pyloric structures of the patients who applied for endoscopic obesity treatment were examined, and the patients were classified as normotonic (NP), hypotonic (HP), and atonic (AP) according to their pyloric structures. PR was applied to those with pyloric structural disorders. Patients with NP were also given the preferred endoscopic treatment (balloon, botulinum toxin, Kanlioz technique). In addition, the pre-procedure fasting blood glucose (FBG) and glycated hemoglobin levels (HbA1c) of the patients were compared with the sixth-month post-procedure status. In order to compare the pyloric structure and other parameters in normal weights with the obese group, a second group of 100 normal-weight (BMI<25) individuals was formed and compared with the study group.

Results: In patients with HP (93 patients) and AP (61 patients), a statistically significant decrease was found between HbA1c and FBG levels before treatment and six months after treatment (p˂0.02, p<0.001, respectively). There was a statistically significant difference in favor of the endoscopic obesity treatment group (EOTG) in terms of pyloric disorder, HbA1c, and FPG levels between the EOTG and the normal weight group (NWG) (p<0.0001).

Conclusion: We recommend using PR as an easy-to-perform, effective, minimally invasive, reproducible, and cost-effective technique that does not require hospitalization.

## Introduction

Obesity can be defined as an excess of body fat [1]. Obesity has emerged as the most critical health problem worldwide, especially in the last 25 years, with the rapid development of information and automation technologies. This technological development resulted in a decrease in labor-intensive jobs, the industrialization of agriculture, the acceleration of migration from rural areas to big cities, and an increase in screen addiction [2]. Hence, obese individuals increased as a natural consequence of a less active lifestyle, high fat and carbohydrate consumption, and overeating habits. The two most essential steps in the prevention of obesity are diet control and exercise [3]. First and foremost, it is essential to limit energy intake and protect musculoskeletal and vascular health by expending energy through exercise [4]. Obesity also impairs quality of life [5]. Several metabolic diseases, particularly “type 2 diabetes mellitus” (type 2 DM), are exacerbated by obesity [6]. Obesity leads not only to type 2 DM but also to a wide range of diseases (cardiovascular disease, joint diseases, etc.) [7]. Having reached such alarming levels globally, obesity is no longer an individual problem but a serious public health issue [8]. Exercising and dieting often remain insufficient for weight loss in obese individuals (BMI >30 kg/m2 ) and those with comorbid metabolic diseases (diabetes, etc.). Those who fail to lose weight effectively with dietary and exercise programs are treated with medical (drug) therapy, endoscopic obesity methods (gastric botox, gastric balloon), and surgical methods (sleeve gastrectomy, gastric bypass, etc.). It is for these reasons that obesity ranks among today's most important health problems [9].

Drawing on past experience, we found the functional structure of the pylorus to be the most crucial factor determining the efficacy of treatment in endoscopic treatment methods for obesity such as intragastric balloon (IGB), intragastric botulinum toxin A (IGBT) injection, and the combination of IGB (+) IGBT (KANLIOZ technique) [10,11]. Having also taken into account the results obtained in our previous studies [11], we sought to minimize pyloric leaks in patients with pyloric leakage by performing filling with “endoscopic intragastric peripyloric injection” (EIPI) in order to provide both obesity treatment and blood glucose regulation in patients with a non-functioning pyloric structure leading to the uncontrolled passage of gastric contents into the duodenum. Because pyloric dysfunction shortens the gastric emptying time and shortens the times of satiety, contact with the high amount of glucose in the duodenum adversely affects the blood sugar mechanism [11]. With this method, it was aimed to prolong the time of satiety and to reduce the amount of glucose in contact with the duodenum per unit of time. We named the procedure performed using EIPI “pyloric revision” (PR). According to the results of our study, we think that PR is a candidate to be the third endoscopic treatment alternative after IGB and IGBT, which are endoscopic bariatric methods, if supported by larger series and the results of other centers.

## Materials and methods

The patients who were referred to our obesity clinic between January 2020 and January 2022 and had a BMI >25 were offered to participate in this study. After all, the patients were informed about obesity treatment methods (diet, surgery, etc.) and the benefits and risks mentioned in the literature about the procedures were explained, and their questions were answered; those who consented were included in the study. Five hundred and twenty-four patients met the inclusion criteria, and 357 (68.1%) patients agreed to participate in this study. They were given at least 24 hours to make a decision, after which their written informed consent was obtained. The patients were also informed that the condition of the pyloric structure (normotonic (NP), atonic (AP), hypotonic (HP)) could only be determined by endoscopy and that PR treatment could be performed if appropriate. All patients were followed after the procedure and had six-month follow-up data. The study was carried out according to the criteria of the Declaration of Helsinki and approval was obtained from the local ethics committee (Istanbul Okan University, approval number 20.04.2023-171).

After obtaining their consent, another group of normal-weight individuals (BMI <25 kg/m2) was formed to evaluate the pyloric structure, fasting blood glucose (FBG), and glycosylated hemoglobin (HbA1c) levels in normal weights. The aim of this group was to compare normal weight and obese patients in terms of the above parameters. When forming this normal weight group (NWG), the first 100 volunteered normal-weight patients were included in the evaluation. The age of the patients in NWG was between 18 and 65 years. The BMI was between 20 and 25 kg/m2 and consisted of patients who were admitted to the hospital for any reason other than obesity and were scheduled for upper gastrointestinal endoscopy. Pyloric structures (after endoscopic evaluation), gender, age, weight, BMI, FPG, and HbA1c values of NWG patients were recorded. Since these patients in NWG were not obese, only upper gastrointestinal endoscopy was performed. Endoscopic obesity treatment was not applied. Endoscopies of these patients were not performed again after six months because PR was not performed.

**Figure 1:**
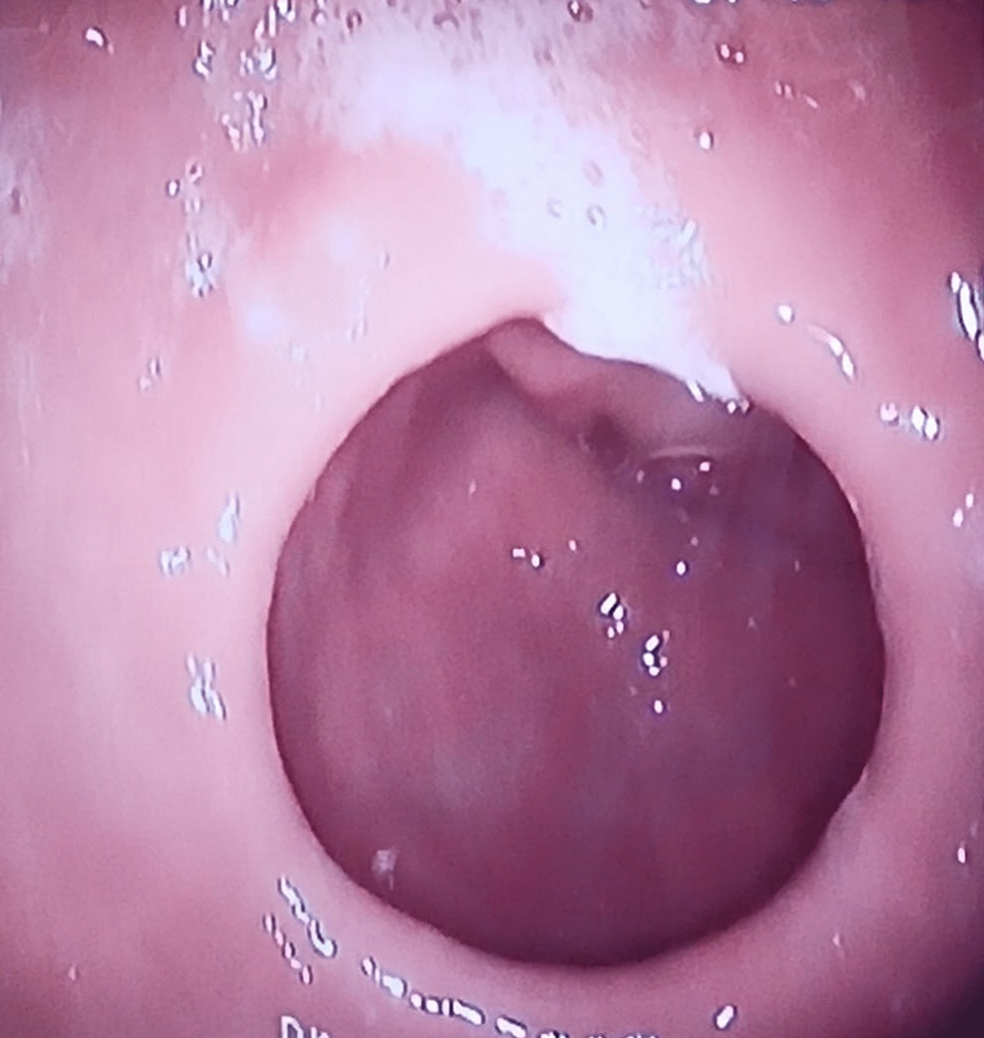
Image of the AP structure before the procedure

**Figure 2:**
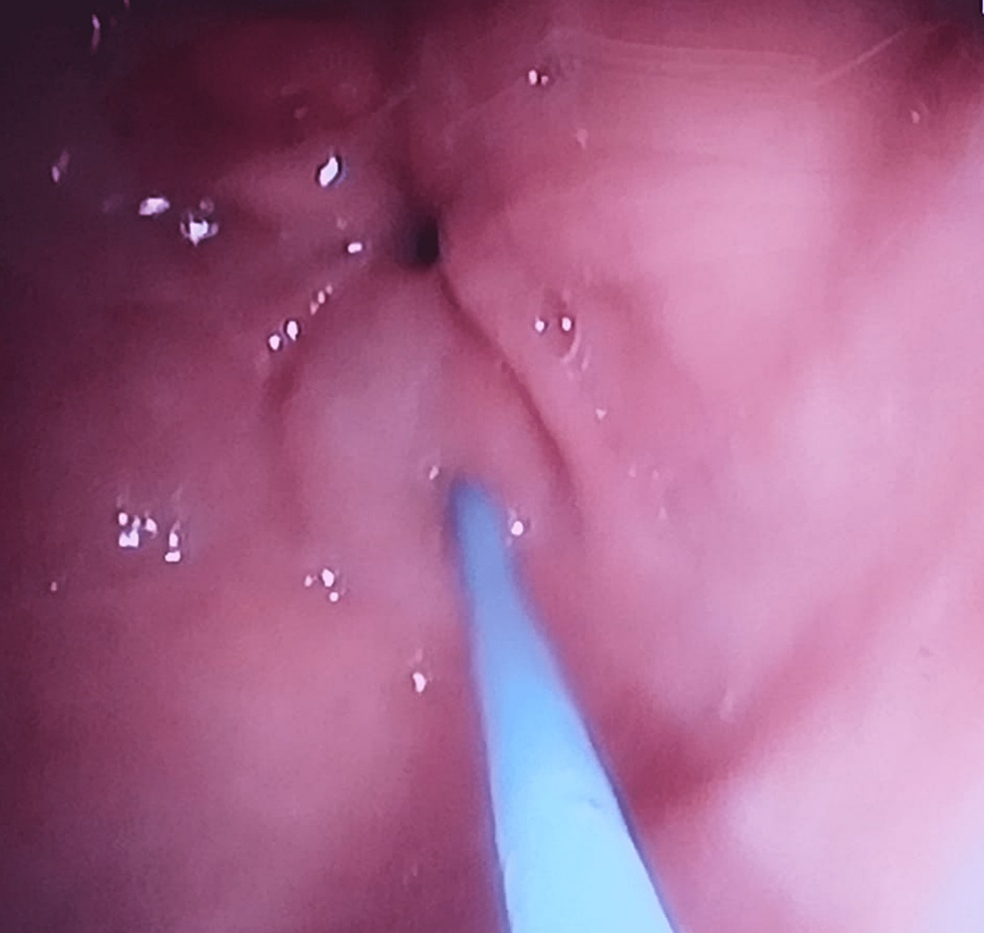
Image of the peripyloric filling procedure performed for PR

**Figure 3:**
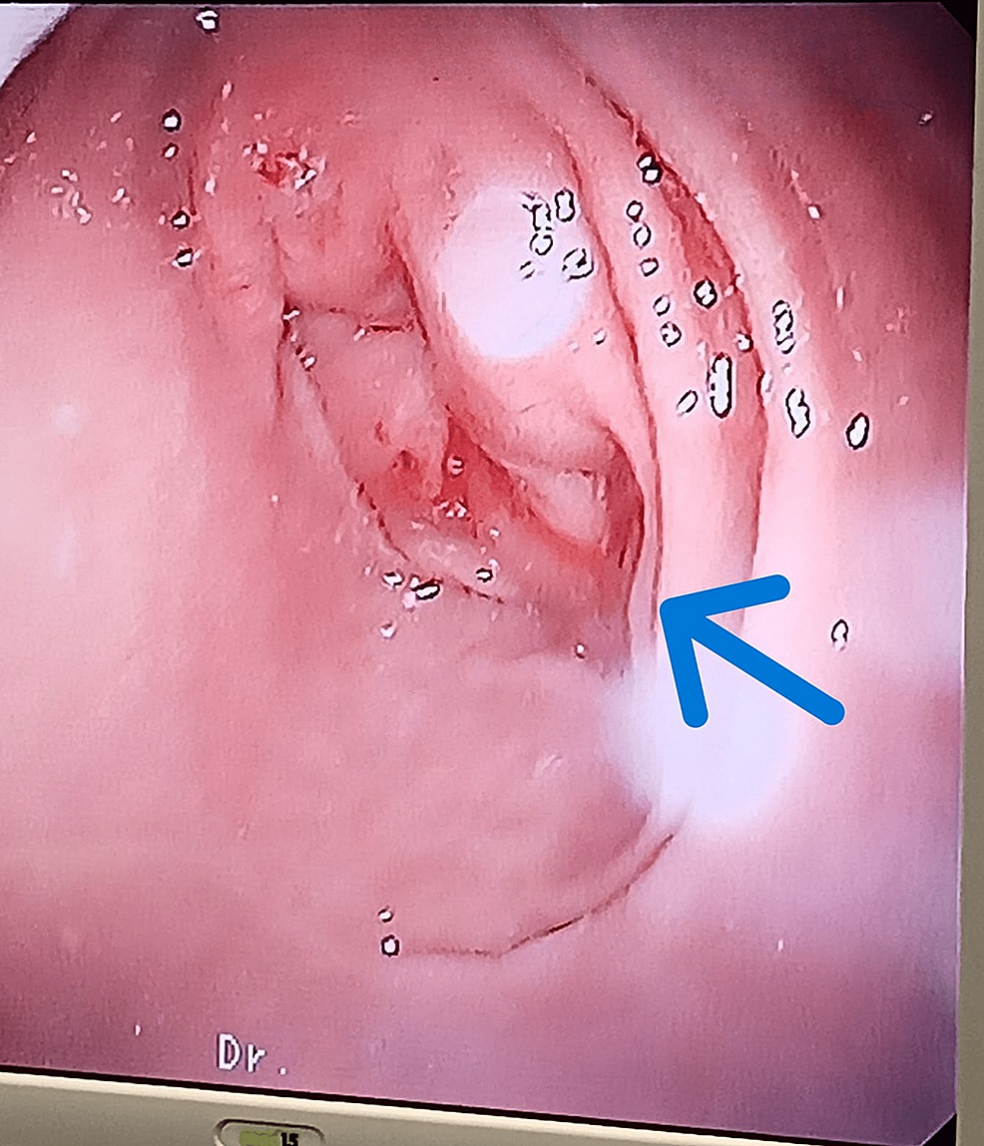
Image of the AP structure before the procedure

The height, weight, BMI, FBG levels, and HbA1c levels were measured and recorded for the "control" and the ''intervention'' groups. In the endoscopic obesity treatment group (EOTG) and the control group, the patients were first evaluated for functional pylorus by endoscopic examination. Upon endoscopic examination, the functional structure of the pylorus was defined as follows: NP if the pylorus closed entirely when mild mechanical stimulation was applied around the pylorus with the endoscope tip after reaching the stomach or spontaneously, HP if it did not close entirely, and AP if there was no contraction in the pylorus despite mechanical stimulation and it was entirely open. Patients with NP in the EOTG received IGBT, IGB, or the KANLIOZ technique (IGBT and IGB at the same time) in the same session because the patients applied to our clinic for the treatment of obesity. In patients with HP and AP, a peripyloric intramuscular injection of 30 cc of 10% NaCl solution was performed endoscopically in the ring 10 mm outside the pylorus at a rate of 5 cc per 60°. Then, the pylorus was closed with a 5-10 mm passage so that the gastric contents could be emptied by gastric peristalsis, and the uncontrolled passage from the stomach to the duodenum was tried to be minimized in all patients (Figures 1-3). All procedures were performed in the same session. PR was performed in patients with abnormal (HP and AP) pyloric function, and in patients with normal pyloric function, other endoscopic procedures described above were performed according to their pre-procedural preferences.

A standard 5-mm sclerotherapy needle was used for endoscopic injections. All procedures were performed in the operating room and under sedation. Following the procedure, the patients remained under observation for two hours and were discharged from the hospital. Furthermore, all patients were referred to a dietician and prescribed a 1200-calorie low-carbohydrate diet. The patients were followed by a dietitian for six months.

Six months after the procedure, the patients' heights, weights, BMI, FBG, and HbA1c levels were measured and recorded. In patients who underwent PR in EOTG, an endoscopic examination was performed six months after the intervention, and the passage status in the pylorus was monitored and recorded.

Statistical method

Values measured before and six months after treatment were analyzed by Student's t-test. Independent group analyses were performed using the independent samples t-test. The distribution of the data within the groups was analyzed with the Kolmogorov-Smirnov test. The pyloric structure was evaluated with EOTG and NWG Mann-Whitney U test. The difference between EOTG and NWG according to HbA1c and FBG levels was evaluated with the Chi-square test of independence. A p-value of ≤0.05 was accepted as statistical significance. All statistical analyzes were performed using SPSS Statistics version 22 (IBM Corp. Released 2013. IBM SPSS Statistics for Windows, Version 22.0. Armonk, NY: IBM Corp.).

## Results

Three hundred and fifty-seven patients were enrolled in the EOTG group, and 221 (61.9%) were female. The mean age was 35.14±12.01 years. The mean weight was 101.13±16.55 kg, and the mean BMI was 36.04±5.80 kg/m2. Of the 357 patients in the EOTG, 154 (43.13%) had HP or AP and underwent PR. Treatments (IGB, IGBT, or KANLIOZ technique) determined before the procedure was applied to 203 (56.87%) patients with NP, depending on the wishes of the patients. The distribution of parameters in the groups was evaluated with the Kolmogorov-Smirnov test. The distribution was also not statistically significant and was homogeneously distributed (p ≥0.05) Table 1 shows the distribution of those in the EOTG by preprocedural pyloric structure, age, weight, BMI, FBG, and HbA1c levels.

**Table 1:** Distribution of patients in the EOTG by their pylorus structure, age, weight, BMI, FBG, and HbA1c before the procedure n: number of patients, BMI: body mass index, FBG: fasting blood glucose, HbA1c: hemoglobin A1C, NP: normotonic, HP: hypotonic, AP: atonic

		NP	HP	AP	Total
Number of patients according to pylorus structure n (%)		203 (56.87)	93 (26.05)	61 (17.08)	357 (100)
Age (year)	Mean	35.93	36.39	34.48	35.14
	Median	34.00	34.00	34.00	34.00
	Std. dev.	10.97	11.64	10.42	12.01
	Min.	19.00	18.00	18.00	18.00
	Max.	58.00	62.00	59.00	62.00
Weight (kg)	Mean	102.80	101.87	99.41	101.13
	Median	97.00	98.00	98.00	98.00
	Std. dev.	15.94	16.26	16.02	16.55
	Min.	83.00	76.00	73.00	73.00
	Max.	139.00	146.00	147.00	147.00
BMI (kg/m^2^)	Mean	36.86	36.26	35.70	36.04
	Median	36.00	35.64	33.91	34.74
	Std. dev.	5.12	5.57	6.16	5.80
	Min.	29.05	23.30	27.00	23.30
	Max.	54.32	55.88	60.40	60.40
FBG before treatment (mg/dL)	Mean	137.76	142.73	144.54	141.62
	Median	112.00	120.00	122.00	116.00
	Std. dev.	59.15	63.59	61.19	61.07
	Min.	78.00	77.00	82.00	77.00
	Max.	310.00	318.00	342.00	310.00
HbA1c before treatment (%)	Mean	6.86	6.91	7.42	7.06
	Median	5.80	5.90	6.40	6.02
	Std. dev.	2.93	2.86	3.17	2.94
	Min.	3.90	3.80	3.80	3.80
	Max.	16.20	16.00	16.80	16.80

There were a total of 154 patients, 93 (60.4%) female and 61 (39.6%) male, in the patient group who underwent pyloric revision (PRWG). The mean age of the PRWG was 35.63±11.78 years.

Table 2 shows the genders, ages, weights, BMI, FBG, HbA1c levels, and pyloric structure of the 100 patients in the NWG. Patients in NWG were homogeneous in terms of age, gender, pyloric structure, HbA1c, and FBG levels (p<0.05).

**Table 2:** Gender, age, weight, BMI, FBG, HbA1c values, and pylorus structure of those in NWG n: number of patients, BMI: body mass index, FBG: fasting blood glucose, HbA1c: hemoglobin A1C, NP: normotonic, HP: hypotonic, AP: atonic

Gender n (%)	
Female	58 (58)
Male	42 (42)
Total	100 (100)
The average age (year)	42.76±10.58
Weight average (kg)	62.84±7.24
BMI average (kg/m2)	23.68±1.04
FBG average (mg/dl)	98.71±14.36
HbA1c average (%)	4.98±1.22
Pylorus structure n(%)	
NP	87 (87)
HP	8 (8)
AP	5 (5)
Total	100 (100)

Table 3 shows the comparison between EOTG and NWG in terms of parameters in the study before obese patients were treated yet. There was a significant difference between EOTG and NWG in terms of pyloric structure (p<0.0001). The difference between both groups in terms of HbA1c and FBG levels was statistically significant (p<0.0001). Both the disorder in the pyloric structure and the blood parameters in the study were higher in EOTG.

**Table 3:** Comparative analysis of NWG and EOTG n: number of patients, NWG: normal weight group, EOTG: endoscopic obesity treatment group, HP: normotonic, AP: atonic, FBG: fasting blood glucose, HbA1c: hemoglobin A1C

	NWG	EOTG	p-value
HP n (%)	8 (8)	93 (26.05)	<0.0001
AP n (%)	5 (5)	61 (17.08)	<0.0001
FBG mg/dL	98.71±14.36	139±18.32	<0.0001
HbA1c (%)	4.98±1.22	6.02±2.84	<0.0001

Table 4 shows the FBG and HbA1c levels of the patients with AP and HP in the PRWG before and six months after treatment. In the patients with HP (93 patients), the mean FBG decreased from a pretreatment level of 142.73±63.59 mg/dL to 122.95±45.43 mg/dL after six months (p˂0.02). In the patients with AP (61 patients), the mean FBG decreased from a pretreatment level of 144.54±61.19 mg/dL to 112.67±32.30 mg/dL after six months (p˂0.01). For all patients with deformed pylorus (154 patients), the mean FBG decreased from a pretreatment level of 143.45±62.45 mg/dL to 118.88±40.93 mg/dL after six months (p˂0.02). In the patients with HP, the mean HbA1c decreased from a pretreatment level of 6.91% to 5.90% after six months (p˂0.03). In the patients with AP, the mean HbA1c decreased from a pretreatment level of 7.42% to 5.91% after six months (p˂0.01). For all patients with deformed pylorus, the mean HbA1c decreased from a pretreatment level of 7.11% to 5.90% after six months (p˂0.02).

**Table 4:** FBG and HbA1c values of patients in PRWG before and six months after PR ↕: it indicates that the p-values represent the relationship between the lower and upper groups, FBG: fasting blood glucose, HbA1c: hemoglobin A1C, HP: hypotonic, AP: atonic

		HP	AP	Total
FBG before treatment (mg/dL)	Mean	142.73	144.54	143.45
	Median	120.00	122.00	121.00
	Std. dev.	63.59	61.19	62.45
	Min.	78.00	82.00	78.00
	Max.	318.00	342.00	342.00
		p˂0.02 ↕	p˂0.01 ↕	p˂0.02 ↕
FBG six months after treatment (mg/dL)	Mean	122.95	112.67	118.88
	Median	102.00	102.00	102.00
	Std. dev.	45.43	32.30	40.93
	Min.	74.00	82.00	74.00
	Max.	284.00	243.00	284.00
HbA1c before treatment (%)	Mean	6.91	7.42	7.11
	Median	5.90	6.40	6.15
	Std. dev.	2.86	3.17	2.99
	Min.	3.80	3.80	3.80
	Max.	16.20	16.80	16.80
		p˂0.03 ↕	p˂0.01 ↕	p˂0.02 ↕
HbA1c six months after treatment (%)	Mean	5.90	5.91	5.90
	Median	5.20	5.60	5.35
	Std.Dev.	2.14	1.86	02.02
	Min.	3.40	3.40	3.40
	Max.	14.20	13.10	14.20

Table 5 shows the weights, BMIs, and weight loss rates of the patients with AP and HP in the PRWG before and six months after treatment. In the HP group, the mean weight decreased from a pretreatment level of 101.87±16.22 kg to 87.75±13.00 kg after six months (p˂0.03). In the AP group, the mean weight decreased from a pretreatment level of 99.41±16.02 kg to 80.36±11.31 kg after six months (p˂0.01). In the PRWG, the mean weight decreased from a pretreatment level of 100.90±16.14 kg to 83.61±12.60 kg after six months (p˂0.02). In the HP group, the mean BMI decreased from a pretreatment level of 36.26±5.57 kg/m^2^ to 30.56±4.37 kg/m^2^ after six months (p˂0.03). In the AP group, the mean BMI decreased from a pretreatment level of 35.70±6.16 kg/m^2^ to 28.83±4.28 kg/m^2^ after six months (p˂0.01). In the PRWG, the mean BMI decreased from a pretreatment level of 36.04±5.80 kg/m^2^ to 29.88±4.40 kg/m^2^ after six months (p˂0.02).

**Table 5:** Weight, BMI, and weight loss rates of FP and HP patients in the PRWG before and six months after treatment ↕: it indicates that the p-values represent the relationship between the lower and upper groups, BMI: body mass index, HP: hypotonic, AP: atonic, PRWG: patient group who underwent pyloric revision

		HP	AP	Total (PRWG)
Weight before treatment (kg)	Mean	101.87	99.41	100.90
	Median	98.00	98.00	98.00
	Std. dev.	16.22	16.02	16.14
	Min.	76.00	73.00	73.00
	Max.	146.00	147.00	147.00
		p˂0.03 ↕	p˂0.01 ↕	p˂0.02 ↕
Weight six months after treatment (kg)	Mean	87.75	80.36	83.61
	Median	83.00	80.00	82.00
	Std. dev.	13.00	11.31	12.60
	Min.	66.00	63.00	63.00
	Max.	123.00	116.00	123.00
Average weight loss rates in six months (%)	Mean	15.54	18.73	16.81
	Median	15.75	17.58	16.58
	Std. dev.	5.37	6.56	6.6
	Min.	2.08	2.33	2.08
	Max.	29.57	36.84	36.84
BMI before treatment (kg/m^2^)	Mean	36.26	35.70	36.04
	Median	35.64	33.91	34.74
	Std. dev.	5.57	6.16	5.80
	Min.	23.30	27.00	23.30
	Max.	55.88	60.60	60.40
		p˂0.03 ↕	p˂0.01 ↕	p˂0.02 ↕
BMI six months after treatment (kg/m^2^)	Mean	30.56	28.33	29.88
	Median	29.74	27.34	29.35
	Std. dev.	4.37	4.28	4.40
	Min.	22.72	22.60	22.60
	Max.	50.13	47.67	50.13

Table 6 shows the designation of patients with HP and AP in the PRWG as normal (≤ 25 kg/m^2^), overweight (25.01-30.00 kg/m^2^), obese (30.01-40.00 kg/m^2^), and morbidly obese (≥ 40, 01 kg/m^2^) according to their BMIs before and six months after treatment as well as their classification by weight loss as less than 5%, between 5.01 and 10.00%, between 10.01 and 15.00%, between 15.01 and 20.00%, and ≥ 20.01%.

**Table 6:** Classification of patients in PRWG according their BMI and weight loss rates before and six months after PR n: number of patients, HP: hypotonic, AP: atonic

		HP n (%)	AP n (%)	Total n (%)
Before treatment	Normal	1 (1.1)	0 (0)	1 (0.6)
	Overweight	8 (8.6)	6 (9.8)	14 (9.1)
	Obese	64 (68.8)	42 (68.9)	106 (68.8)
	Morbid obese	20 (21.5)	13 (21.3)	33 (21.4)
	Total	93 (100)	61 (100)	154 (100)
Six months after treatment	Normal	6 (6.5)	6 (9.8)	12 (7.8)
	Overweight	43 (46.2)	38 (62.3)	81 (52.6)
	Obese	41 (44.1)	16 (26.2)	57 (37.0)
	Morbid obese	3 (3.2)	1 (1.6)	4 (2.6)
	Total	93 (100)	61 (100)	154 (100)
Weight loss rates	≤5%	4 (4.3)	2 (3.3)	6 (3.9)
	5.01-10.00%	6 (6.5)	2 (3.3)	8 (5.2)
	10.01-15.00%	33 (35.5)	13 (21.3)	46 (29.9)
	15.01-20.00%	31 (33.3)	21 (34.4)	52 (33.8)
	≥20.01%	19 (20.4)	23 (37.7)	42 (27.3)
	Total	93 (100)	61 (100)	154 (100)

Table 7 shows the classification of patients with HP and AP in the PRWG as normal (5.6% ≤), pre-diabetic (5.7-6.4%), and diabetic (6.5% ≥) according to their HbA1c values before and six months after treatment.

**Table 7:** Classification of patients in HP and AP groups according to HbA1c values before and six months after treatment n: number of patients, HP: hypotonic, AP: atonic

		HP n (%)	AP n (%)	Total n (%)
Before treatment	Normal	43 (46.2)	23 (37.7)	66 (42.9)
	Prediabetes	12 (12.9)	10 (16.4)	22 (14.3)
	Diabetes	38 (40.9)	28 (45.9)	66 (42.9)
	Total	93 (100)	61 (100)	154 (100)
Six months after treatment	Normal	53 (57.0)	34 (55.7)	87 (56.5)
	Prediabetes	12 (12.9)	10 (16.4)	22 (14.3)
	Diabetes	28 (30.1)	17 (27.9)	45 (29.2)
	Total	93 (100)	61 (100)	154 (100)

Six months after PR, follow-up endoscopy could be performed only in 24 out of the 61 patients with AP. The follow-up endoscopy showed that three of the 24 patients with AP had complete pyloric leakage, 11 had partial leakage, and 10 had no leakage. Follow-up endoscopy was performed in 31 out of the 93 patients with HP six months after PR. In the 31 patients with HP, follow-up endoscopy showed that the therapeutic effect of the treatment had utterly disappeared in five patients, while the treatment was partially effective in seven patients and utterly effective in 19 patients.

## Discussion

Several factors are involved in the development of obesity [12,13]. In particular, the mechanisms of hunger and satiety are of paramount importance [14]. In all treatments for obesity, the objectives pursued include eating less, reducing stomach volume, prolonging gastric emptying time, and reducing food absorption by bypassing part of the intestines. There are two effective mechanisms in the formation of satiety, the first being the increase in blood glucose level and the other being the fullness of the stomach and the pressure of food on the stomach wall [15-17]. Various parameters such as eating habits, psychic and endocrine factors, the quality of food consumed, the speed of eating, dietary diversity, the proportion of ready-to-eat foods consumed, concomitant diseases, and cultural factors play an active role in the formation of obesity [18-20].

The functional anatomical structure of the pylorus, which controls the gastric outlet, is crucial in the formation of hunger and satiety mechanisms, both in the rise of blood glucose and in the formation of gastric fullness. In AP, the duration of food in the stomach is short, and food passes uncontrollably and rapidly into the duodenum before the digestive processes in the stomach are completed. The rapid passage of food from the stomach to the duodenum and the rapid absorption of carbohydrates in the intestines cause a rapid rise in blood glucose levels. The endocrine system reacts to the rapid rise in blood glucose levels by secreting a significant amount of insulin, which results in a sudden drop in blood glucose levels. A rapid rise in blood glucose levels followed by a sudden drop triggers the hunger mechanism, leading people to seek food, especially carbohydrates, at shorter intervals and at much greater intensity. Thus, the excess energy intake exceeding the daily need causes obesity. Equally important as the sudden rise and drop in blood glucose levels, people with AP do not feel as full as those with NP, no matter how much they eat, because their stomachs are constantly leaking, and they will never feel as full as people with NP. For these reasons, we believe that the functional structure of the pylorus needs to be taken into account in the treatment of obesity. In our previous studies, among patients who underwent IGB and IGBT, patients with NP lost the most weight, followed by those with HP and then AP [10,11].

After the studies on the effect of the structure of the pylorus on weight gain and weight loss, the literature on interventions to correct this problem was reviewed. No study was found to correct the pyloric structure applied to the prepyloric region. However, it was decided to use a 10% saline solution, which is injected into the stomach for the treatment of obesity [21] and is also used in the treatment of rectal prolapse [22,23]. Afterward, the technique described in the above sections was developed.

There are many papers in which the results of obesity treatments are diametrically opposed. We believe that if the studies describing such contradictory results were reviewed with the structure of the pylorus in mind, contradictory results of this magnitude would not be observed. Topazian et al. stressed that botulinum toxin A (BTA), to be performed under endoscopic ultrasound guidance of IGBT treatment, would be highly effective in slowing gastric emptying and promoting weight loss [24]. In their meta-analysis, Yen et al. reported that 200 IU BTA was remarkably effective for weight loss [25]. Bustamente et al., however, reported in their two different studies, one of which was a meta-analysis, that IGBT treatment was ineffective for weight loss [26].

Because PR is a minimally invasive method, it has advantages such as the possibility of re-administration. Similarly, IGBT is an advantageous method in the treatment of obesity because of its ease of administration and lack of severe side effects. Although IGB is also highly effective in well-selected groups of patients, PR and IGBT appear to be more advantageous, given the intolerances and the need for a second procedure to remove the balloon. We believe that PR deserves further research given its advantages, such as the minimal risk of mortality and morbidity, ease of administration, affordability, lack of labor loss, and effectiveness. In terms of ease of administration and effectiveness in treating obesity, PR appears to be equal to or more advantageous than other endoscopic methods for treating obesity. Kaya et al. demonstrated a 9-kg weight loss and a 3-unit decrease in BMI after an average of six months of IGBT treatment [27]. Hernandez et al. reported achieving a mean weight loss of 10.7 kg in six months with IGB treatment [28]. Our study resulted in a mean weight loss of 17.29 kg over a period of six months. The mean rate of weight loss in the PRWG was 16.81±6.06%. Rollnik et al. reported a mean weight loss of 8.9% from baseline weight with IGBT treatment [29].

In addition to its effectiveness in weight loss in obesity, PR is also vital in diabetes regulation. As is known, the rate of diabetes increases with obesity. Dietz et al. reported in their study that the rate of DM type 2 in adults increased significantly with obesity [30]. Furthermore, in their study, Osinski et al. reported that diabetes increased with obesity [7]. Comparing FBG and HbA1c levels before and six months after PR, the differences in all groups were statistically significant in our study. PR ensures both blood glucose regulation and weight loss. We suggest that the dietary program applied in conjunction with PR was also effective in both weight loss and the decrease in FBG and HbA1c levels.

The follow-up endoscopy performed six months after PR showed no change in the effectiveness of the treatment in the majority of patients, while, in some, there was a partial regression, and, in others, the effectiveness had regressed entirely. We continue our much more comprehensive study programs on factors affecting the continuity of effectiveness and the evaluation of alternatives for filling.

In the EOTG, the mean BMI was 36.04±5.80 kg/m^2^ and 43.13% of the patients had HP or AP. In the NWG, BMI was 23.68±1.04 kg/m^2^, and 13% of the patients had HP or AP. The difference between the two groups was statistically significant, which requires further and broader research (p˂0.0001). Furthermore, the differences between the EOTG and the NWG with respect to FBG and HbA1c values were also significant (p˂0.0001). The differences between the groups indicate that obesity is a severe public health problem in all its aspects. Given the rate of increase of obesity and the population it affects worldwide, it is evident that it is challenging to find a solution with treatment methods. To the extent that a strategy can be created to address obesity as a public health issue, obesity rates can be reduced.

Endoscopic and subjective evaluation and classification of pyloric laxity is a limiting factor in our study. Furthermore, in the study, there was no control cohort in which participants did not receive the pyloric injection. As all participants received strict dietary therapy after pyloric injection, the beneficial effects observed in the current study could potentially be attributed to dietary therapy. If the results are supported by other studies that provide objective motility assessment and studies that eliminate other limitations, the effects of PR can be determined more clearly.

## Conclusions

As a relatively new technique that needs to be supported by further research, PR yields promising and encouraging results, and we recommend its use for the treatment of patients with AP and HP who seek to lose weight. However, more research is needed to determine the potential of this treatment option.
